# Asymmetric Electrostrain/Electrobending in Piezoelectric Ceramics: Role of Defect Dipoles or Oxygen Vacancies

**DOI:** 10.1002/advs.75649

**Published:** 2026-05-10

**Authors:** Jie Wang, Geng Huangfu, Hongjie Zhang, Tiannan Yang, Zhenhua Ma, Jingsheng Chen, Yun Liu, Yiping Guo

**Affiliations:** ^1^ State Key Laboratory of Metal Matrix Composites School of Materials Science and Engineering Shanghai Jiao Tong University Shanghai China; ^2^ Department of Materials Science and Engineering National University of Singapore Singapore Singapore; ^3^ Interdisciplinary Research Center School of Mechanical Engineering Shanghai Jiao Tong University Shanghai China; ^4^ Research School of Chemistry / College of Science the Australian National University Canberra Australia

**Keywords:** defect dipole, electrobending, electrostrain, oxygen vacancy, piezoelectric ceramic

## Abstract

Atomic‐scale defects in piezoelectric ceramics significantly influence their macroscopic electric field‐induced deformation responses, which are critical for developing advanced piezoelectric actuators. Although the recently revealed giant electrostrain and electrobending have been attributed to defect dipoles or oxygen vacancies, deciphering their precise atomic configurations and specific roles remains a great challenge. In this study, the microscopic defect configurations and mesoscopic domain switching behaviors of different piezoelectric ceramics with macroscopic asymmetric electrostrain/electrobending are visualized. We demonstrate that the long‐range migration of oxygen vacancies alters surface domain switching and induces poling‐independent electrobending deformation in BaTiO_3_ ceramics, while the formation of defect dipoles can effectively suppress such electrobending and cause poling‐dependent asymmetric electrostrain. Notably, we found that the defect dipoles with a continuous gradient distribution will undergo self‐alignment in (K, Na)NbO_3_‐based ceramics, thereby elucidating the origin of poling‐direction‐independent electrobending in piezoelectric ceramics with volatile elements. These findings mark a milestone in understanding the mechanism underlying giant defect‐mediated electro‐deformation, settle widely debated controversy about electrobending, and open new avenues for the rational design of advanced piezoelectric ceramics.

## Graphical abstract

Defect‐configurational origins of the asymmetric apparent electrostrain are revealed in different piezoelectric ceramics via atomic‐scale visualization of defect configurations. Migration of oxygen vacancies leads to the electrobending effect in N2‐sintered BaTiO_3_, while defect dipoles in Ba_0.99_TiO_2.99_ generate true asymmetric electrostrain without bending effect. In volatile Sr‐doped (K, Na)NbO^3^ ceramics, gradient distribution of defect dipoles drives self‐alignment, resulting in poling‐independent electro‐induced deformation.

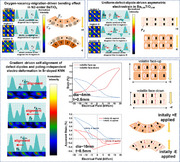

## 1 Introduction

Oxygen vacancies and their associated charged defects can impart beneficial physical and chemical properties to a broad range of functional materials [1, 2, 3, 4, 5, 6, 7, 8]. For example, oxygen vacancies play pivotal roles in La_3_Ni_2_O_7__−__δ_ superconductors [9], ionic conduction in solid oxide fuel cells [10], modulation of oxide‐supported catalytic processes [11], strengthening and toughening of piezoelectric ceramics [12], and formation of defect dipoles that enhance energy storage performance in ferroelectrics [13]. In piezoelectric ceramics, defect engineering has long been utilized to tailor “soft” or “hard” characteristics for improving dielectric and piezoelectric performance or enhancing mechanical quality factor, respectively [14, 15, 16, 17]. Recently, giant asymmetric electrostrain has been reported in numerous lead‐free piezoelectric ceramics containing volatile elements, such as (K, Na)NbO_3_ (KNN) and (Na, Bi)TiO_3_ (NBT), attributed primarily to the formation of defect dipoles or the migration of isolated oxygen vacancies [1, 2, 4, 5, 6, 8, 18, 19]. However, due to the structural diversity and complexity of defect configurations in piezoelectric ceramics, the precise presence and individual contributions of specific defect configurations to electrostrain behavior have yet to be conclusively established.

Our previous work showed that the distinctive giant asymmetric true strain–electric field (S‐E) responses should be attributed to the interplay between domain switching and defect dipoles $V_{K/Na}^{\prime }$‐$V_{O}^{\cdot \cdot }$, in which the vacancies are introduced via volatilization during sintering [1]. Meanwhile, a gradient distribution of defect dipole arising from volatilization or nonstoichiometric composition design would induce electrobending deformation [2, 3]. The pronounced asymmetric S–E responses in KNN and NBT systems were also interpreted as solely electrobending effects, wherein the direction of bending deformation is independent of the poling direction [5, 6, 7, 8]. For instance, Tian et al. suggested that the opposing spontaneous alignment of surface defect dipoles induces large electrobending deformation [5]. He et al. attributed such electrobending effect to the interplay between the surface effect and non‐180° ferroelastic domain switching [7]. Das Adhikary et al. proposed that electric‐field‐induced migration of isolated oxygen vacancies can influence domain switching at vacancy‐modified surfaces, leading to bending deformation of the piezoelectric ceramic with small and symmetric true S–E responses [8]. The absence of consensus regarding the origin of defect‐mediated electrobending/giant electrostrain primarily stems from an insufficient understanding of defect configurations, particularly defect dipoles, significantly impeding fundamental insights and the ability to utilize defects to tailor material properties.

**FIGURE 1:**
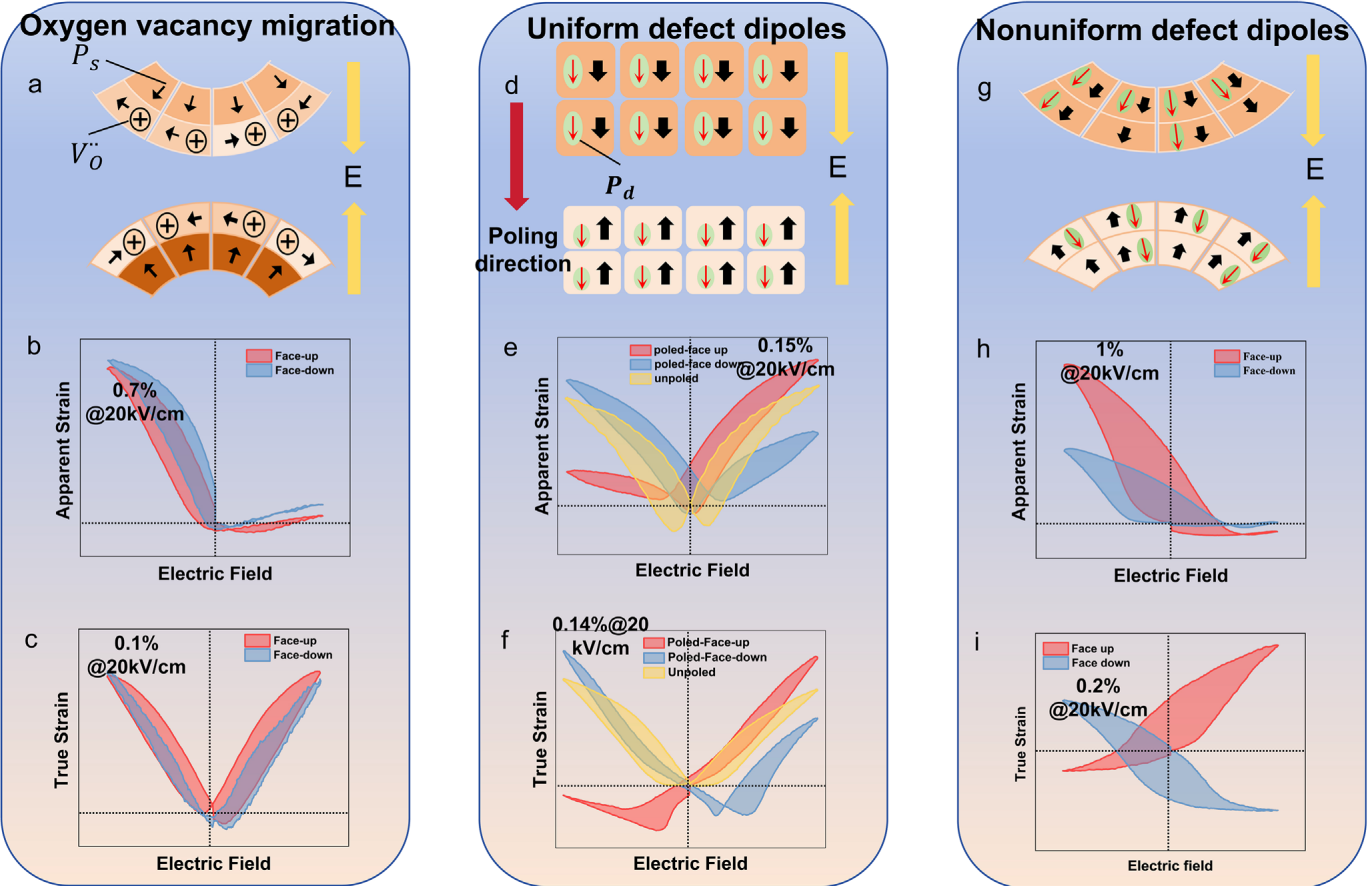
Electric‐field‐induced deformation arising from different defect states. (a) Schematic illustration of electric‐field‐induced bending deformation caused by the migration of oxygen vacancies; (b) Apparent strain–electric field (S–E) curve resulting from electrobending induced by oxygen‐vacancy migration; (c) True electric‐field‐induced strain curve of a sample exhibiting oxygen‐vacancy migration; (d) Schematic illustration of asymmetric electric‐field‐induced strain generated by uniformly distributed, oriented defect dipoles (the orientation of defect dipoles is realized during high‐temperature poling process); (e) Apparent electric‐field‐induced strain response before and after polarization of uniformly distributed defect dipoles; (f) True strain before and after polarization of uniformly distributed defect dipoles; (g) Schematic illustration of bending deformation induced by non‐uniformly distributed defect dipoles (the orientation of defect dipoles is realized due to defect gradient); (h) Apparent electric‐field‐induced strain curve arising from electrobending caused by non‐uniformly distributed defect dipoles; (i) True electric‐field‐induced strain curve associated with non‐uniformly distributed defect dipoles.

In this study, piezoelectric ceramics with different defect states were synthesized, and their microscopic defect configurations, mesoscale ferroelectric behaviors, and macroscopic electric‐field‐induced deformations were systematically investigated. BaTiO_3_ ceramics sintered under an N_2_ atmosphere (denoted as BT‐N) contain a high concentration of unpaired oxygen vacancies, which undergo migration under an applied electric field and thereby induce electric‐field‐driven bending deformation (Figure 1a). Such bending leads to asymmetric apparent strain–electric field (S‐E) curves (the macroscopically measured strain arising from bending is referred to as “apparent strain,” whereas the strain generated by the inverse piezoelectric effect of the material is defined as “true strain” and denoted as strain for short), whereas the true strain curve remains symmetric after poling (Figure 1b,c). In contrast, non‐stoichiometric Ba_0_._99_TiO_2_._99_ (denoted as B99T) ceramics contain 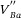
‐$V_{O}^{\cdot \cdot }$ defect dipoles, in which oxygen vacancy migration is suppressed, leading to a polarization‐direction‐dependent asymmetric strain response (Figure 1d–f). In volatile (K, Na)NbO^3^‐based ceramics, we observed self‐alignment of $V_{K/Na}^{\prime }$‐$V_{O}^{\cdot \cdot }$ defect dipoles induced by the continuous gradient distributions of 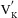/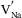 and $V_{O}^{\cdot \cdot }$, which is also supported by the phase‐field simulation and first‐principles calculation results. This alignment results in a poling‐direction‐independent electrobending effect (Figure 1g,h), which can be readily confused with electrobending caused by oxygen‐vacancy migration. However, in this case, the true strain of the ceramic is also asymmetric and can be inverted by flipping the sample (Figure 1i), which is fundamentally distinct from the electro‐induced deformation arising from oxygen‐vacancy migration. These results clarify electric‐field‐induced behavior of different defect configurations and the origin of large electro‐deformation in piezoelectric ceramics, offering crucial insights for the design of advanced piezoelectric ceramics.

## 2 Results and Discussion

### 2.1 Distinguishing Roles of Unpaired Oxygen Vacancies and Defect Dipoles in the Electrostrain Response of Piezoelectric Ceramics

**FIGURE 2:**
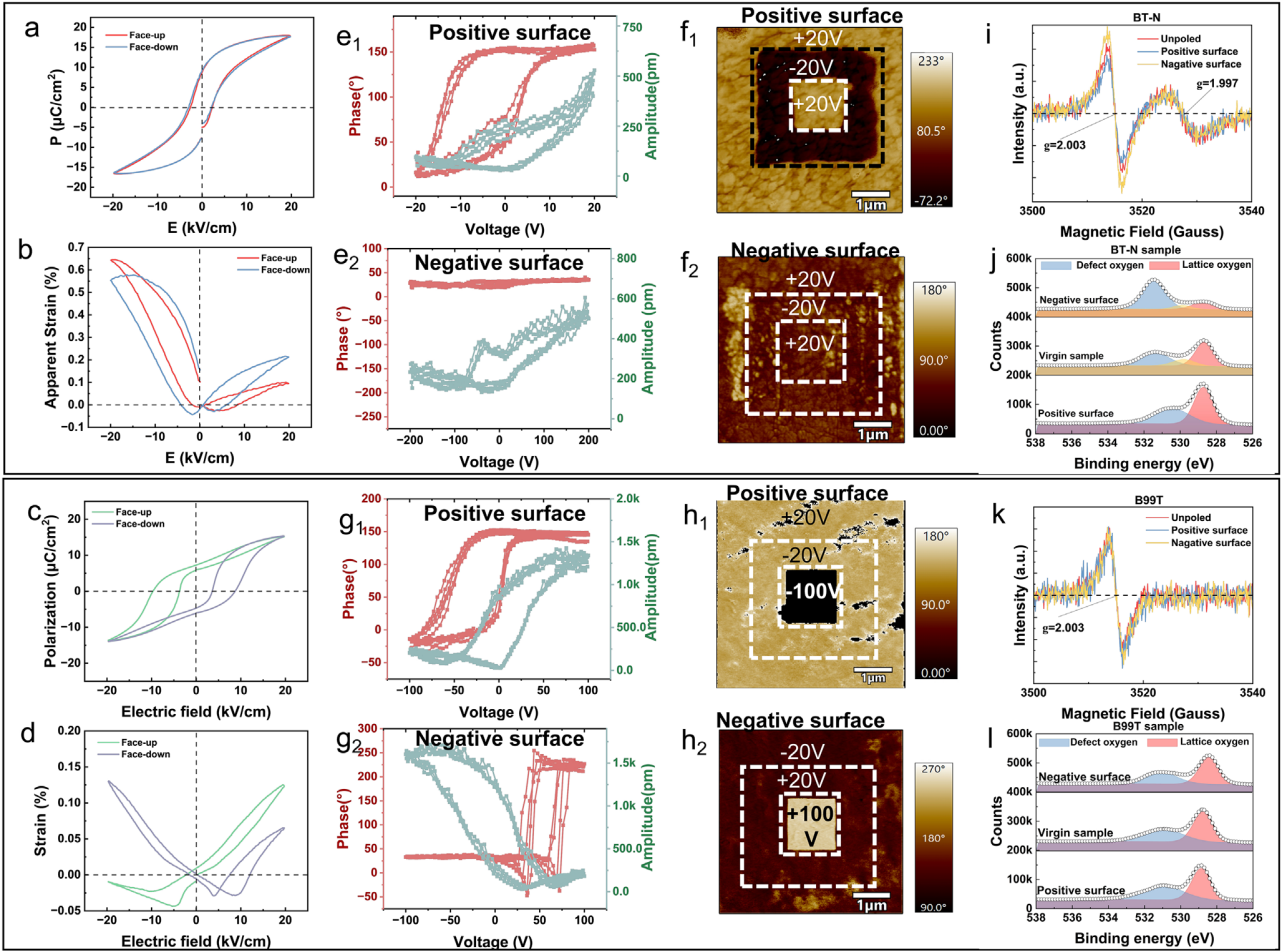
(a, b) Polarization‐Electric field (P‐E) loops and apparent strain‐electric field (S‐E) curves of BT‐N sample; (c, d) P‐E loops and S‐E curves of B99T sample; (e_1_‐e_2_) phase angle and piezoelectric amplitude‐voltage curves of the surfaces of poled BT‐N sample measured utilizing Piezoelectric force microscopy (PFM); (f1‐f2) domain writing results for positive surface and negative surface of BT‐N sample; (g_1_‐g_2_) phase angle and piezoelectric amplitude‐voltage curves of the surfaces of poled B99T sample measured utilizing PFM; (h1‐h2) domain writing results for positive surface and negative surface of B99T sample; (i) Electron paramagnetic resonance (EPR) spectroscopies of virgin (unpoled), positive and negative surfaces of BT‐N; (j) X‐ray photoelectron spectroscopy (XPS) spectra of O1s for virgin, positively poled, and negatively poled surfaces of BT‐N; (k) Electron paramagnetic resonance (EPR) spectroscopies of virgin (unpoled), positive and negative surfaces of B99T; (l) X‐ray photoelectron spectroscopy (XPS) spectra of O1s for virgin, positively poled, and negatively poled surfaces of B99T.

We first investigated the apparent electro‐deformation response of the BT‐N ceramics. The P–E hysteresis loops of the BT‐N ceramics do not exhibit any internal bias field even after poling (Figure 2a). In contrast, the apparent electric‐field‐induced strain curves of the 0.3‐mm‐thick BT‐N specimen (10 mm in diameter) are asymmetric with a giant strain value of 0.65% at 20 kV cm^−1^, and their shapes remain unchanged upon flipping the sample, as shown in Figure 2b. The unique apparent electrostrain responses of BT‐N are considered as a typical feature of electrobending deformation [2, 7]. The measurement process of the apparent S‐E curves is illustrated in Figure S1b. The discrepancy in contact area between the upper and lower clamps results in larger/smaller displacements when the sample bends convexly/concavely. A Doppler laser scanning vibrometer was used to visualize the deformation of the BT‐N samples under alternating electric fields, as shown in Video S1. Appreciable bending was observed under ±20 kV cm^−1^ alternating electric fields for samples with a thickness of 0.3 mm, confirming that the apparent asymmetric electrostrain is associated with bending deformation. By performing electric‐field‐induced strain measurements using fixtures and samples of different geometries, we decoupled the bending‐induced apparent strain from the true strain in BT‐N ceramics. The results show that the true electric‐field‐induced strain curves remain symmetric, indicating no contribution of defect dipoles in BT‐N samples (see Note S1). It is also found that the thinner samples exhibit larger electro‐induced bending and correspondingly higher measured apparent electrostrain values (Figure S2b–d), while the 1 mm‐thick sample (the bending effect can be negligible at this thickness) exhibits a symmetric strain response, which is often observed in piezoelectric ceramics with a bending effect. The thickness‐dependent apparent strain originating from oxygen vacancy migration is quantitatively analyzed in Note S2.

The B99T ceramics exhibit a completely different electro‐deformation response. Doppler laser scanning vibrometer measurements show that the poled 0.3‐mm‐thick B99T sample (10 mm in diameter) undergoes relatively uniform piezoelectric strain with negligible bending deformation under AC electric fields (Video S2). The P–E hysteresis loop of the poled B99T sample displays a pronounced internal bias field (Figure 2c), and the strain curve of the B99T sample exhibits a pronounced asymmetry, consistent with the presence of the internal bias field (Figure 2d). This is commonly attributed to the effect of aligned defect dipoles. The strain curve exhibits mirror reversal upon reversal of the poling direction when the sample is flipped from face‐up to face‐down. This poling‐direction‐dependent asymmetric electrostrain response is observed across varied measurement fixtures (Note S1). As the sample thickness varies, the strain values of B99T ceramics, both before and after poling, remain essentially unchanged, confirming that the apparent asymmetric electrostrain in the B99T sample represents true strain rather than bending deformation (Figure S2f,h). The internal bias field becomes more pronounced in thinner samples (Figure S2g), owing to the enhanced alignment of surface‐near defect dipoles during the poling process [20].

To reveal the origin of the different electro‐deformation in B99T and BT‐N, we employed piezoresponse force microscopy (PFM) to investigate the surface domain switching behavior. For unpoled BT‐N samples, the amplitude‐voltage and phase‐voltage curves show that the surface domain switching is not restricted (Figure S3 c1). Domain writing results also confirm that the surface domain can be completely reversed after applying +20 V or −20 V voltage (Figure S3 e1). For the poled BT‐N sample, the surface domains near the positive electrode show similar amplitude and phase curves to the unpoled samples, and the domains can be completely reversed after applying domain writing voltages (Figure 2 e1,f1). However, the surface domain near the negative electrode shows a negligible phase response even at ±200 V, a tenfold higher voltage, accompanied by significantly constrained domain switching after applying domain writing voltage (Figure 2 e2,f2). This can be ascribed to oxygen‐vacancy migration [8, 21], which results in different oxygen‐vacancy concentrations at two opposite surfaces. In the B99T sample, the surface ferroelectric domain behavior prior to poling is similar to that of BT‐N (Figure S4 c1,e1); however, after poling, the amplitude–voltage and phase–voltage curves measured at the positive and negative surfaces exhibit pronounced asymmetry that is compatible with the macroscopic P–E loops and S–E curves (Figure 2 g1–g2). This asymmetry should be attributed to an asymmetric ferroelectric response induced by oriented defect dipoles, as aligned 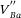
‐$V_{O}^{\cdot \cdot }$ can generate an internal bias field that alter domain switching process. The domain‐writing results further confirm that ferroelectric domain switching at both the positive and negative surfaces is enhanced when driven along the poling direction, but is suppressed when driven against it (Figure 2 h1,h2), demonstrating that the asymmetric electrostrain response of the B99T sample arises from the interaction between oriented defect dipoles and ferroelectric domains.

In order to confirm the migration behavior of oxygen vacancies in BT‐N and B99T samples, electron paramagnetic resonance (EPR) spectroscopy and X‐ray photoelectron spectroscopy (XPS) were conducted (Figure 2i–l). EPR and XPS results both indicate that the oxygen vacancies locally accumule on the surface near the negative electrode in the BT‐N sample (Figure 2i), which results in the different domain switching behavior near the positive surface and the negative surface. In contrast, such migration of oxygen vacancies is not observed in B99T samples. It should be noted that EPR detects a signal from Ti^3^^+^ in BT‐N (g = 1.997) [22], which indicates that the oxygen vacancies in BT‐N donate two electrons that are mainly localized as Ti^3^^+^ small polarons at neighboring Ti sites [23]. Under such conditions, oxygen vacancies in BT‐N are unlikely to form stable defect‐dipole complexes and therefore, exhibit relatively high mobility [24].

**FIGURE 3:**
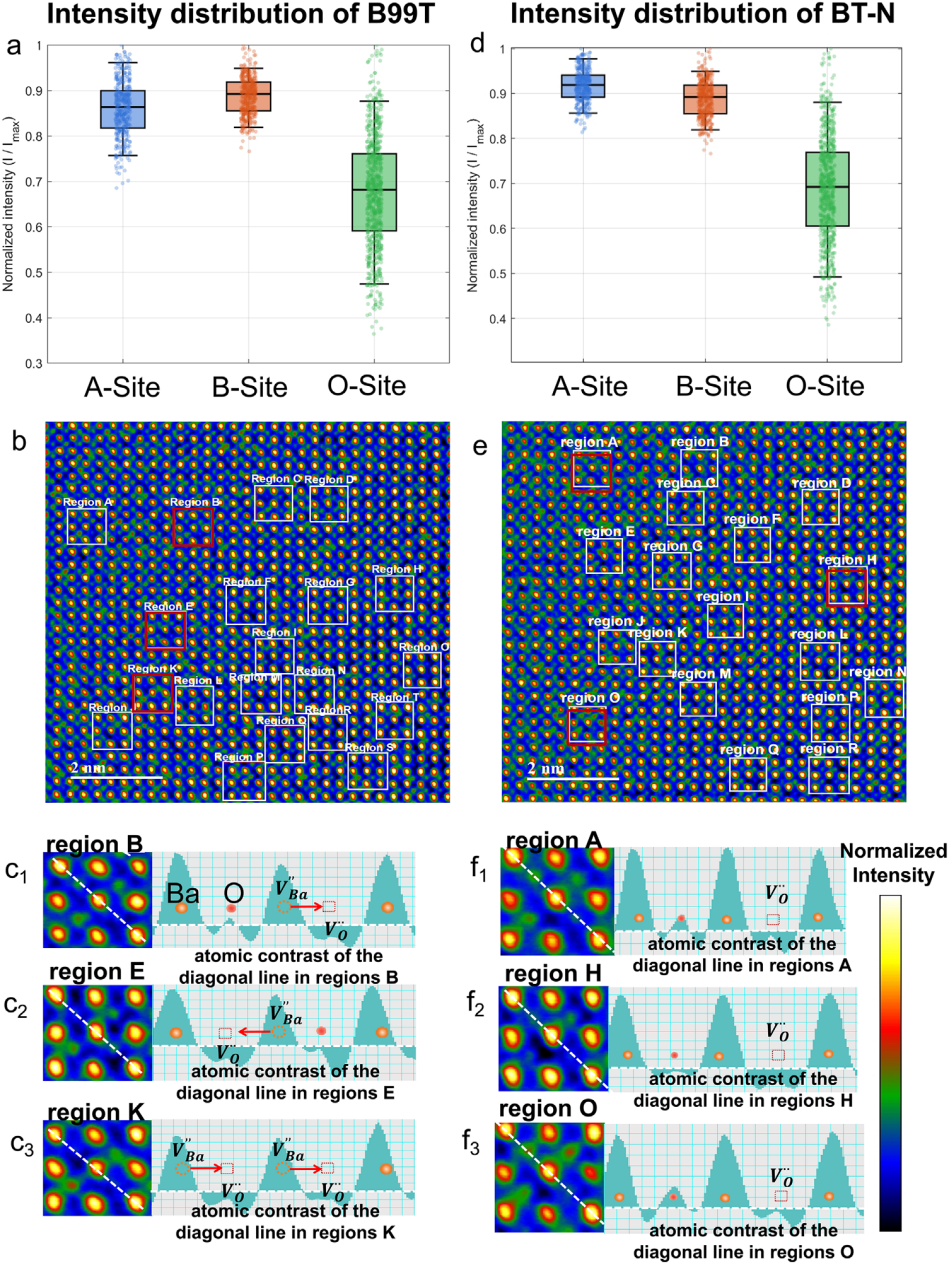
(a) scatter plots and boxplots of the intensity distributions at the A‐, B‐, and O‐sites in the B99T sample; (b) colored iDPC image of B99T sample; (c1‐c3) enlarged planar atomic contrast of regions B, E, and K in Figure 3b and corresponding line atomic contrast of the diagonal lines (from left to right). Certain Ba and O sites exhibit distinctly lower contrast compared with neighboring atomic columns (identified by dashed boxes), and consistently appear in coupled pairs, indicating the existence of defect dipoles with the projection of the <011> direction. (d) Scatter plots and boxplots of the intensity distributions at the A‐, B‐, and O‐sites in the BT‐N sample; (e) colored iDPC image of BT‐N sample; (f1‐f3) enlarged planar atomic contrast of regions A, H, O in Figure 3e and corresponding line atomic contrast of the diagonal lines (from left to right).

To further visualize the atomic‐scale defect configuration, integrated differential phase contrast scanning transmission electron microscopy (iDPC‐STEM) was employed. Since the internal potential distribution is directly related to the type and precise positions of atoms within the sample, this method enables the visualization of individual atomic positions (Figure S5). For iDPC‐STEM characterization, all samples were thinned to 10–20 nm to minimize atomic column overlap and enable better visualization of oxygen vacancies. We first investigated the contrast distribution at the A‐, B‐, and O‐sites in the B99T sample along the [001] zone axis, and the normalized intensity distributions of the A‐, B‐, and O‐sites are shown in Figure S6a–c. For the B99T sample, the iDPC images reveal pronounced contrast fluctuations for both A‐site cations and oxygen atoms, while the B‐site cations exhibit much smaller contrast fluctuations, indicating the existence of A‐site vacancies and oxygen vacancies in the sample (Figure 3a). The dispersed contrast distributions at the A‐ and O‐sites, together with the much narrower distribution at the B‐site, indicate that the observed contrast variations are unlikely to stem from extrinsic artifacts such as local thickness fluctuations at the atomic scale of a few atoms or beam‐induced damage. If such effects dominated, the heavier B‐site columns would be expected to exhibit stronger contrast perturbations than the lighter oxygen columns. To further eliminate the influence of these interfering factors on the intensity distribution and to more accurately examine the microscopic defect configurations, several regions containing paired O‐site vacancies and A‐site vacancies were selected from the iDPC‐STEM image and enlarged for more detailed line contrast analysis (Figure 3b). Certain A‐ and O‐sites exhibit distinctly lower contrast compared with neighboring atomic columns (identified by boxes), and they are spatially adjacent to each other. Magnified atomic‐contrast maps around these defect sites, together with the corresponding contrast profiles extracted along the diagonal directions, are presented in Figure 3c1–c3 as a representative example. These results provide a clear visualization of A‐site vacancies and their adjacent O‐site vacancies aligned along the ⟨011⟩ direction, where both sites exhibit reduced intensity. It should be noted that each “atomic site” in an iDPC image corresponds to an atomic column arising from the superposition of multiple unit cells along the beam direction; therefore, two neighboring sites in the 2D projection are not necessarily adjacent in 3D space. However, if these vacancies were merely isolated (i.e., not coupled into defect dipoles), they would be unlikely to generate the pronounced internal bias manifested by the shifted P–E hysteresis loops (Figure 2c) and the distinctive PFM domain‐writing behavior (Figure 2h 1,2). Therefore, the observed adjacent vacancy sites can be reasonably regarded as candidate locations for 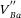
‐$V_{O}^{\cdot \cdot }$ defect‐dipole configurations. Figure S7 illustrates the abundant possible positions of 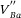
‐$V_{O}^{\cdot \cdot }$ defect dipoles in the B99T sample, which serve as the dominant defect configuration. By comparison, the BT‐N sample exhibits large contrast fluctuations only for oxygen atoms, whereas the contrast fluctuations of both A‐site and B‐site cations are minimal, indicating that the dominant defect configuration in BT‐N consists of unpaired oxygen vacancies, with low concentrations of A‐site and B‐site vacancies (Figure 3d; Figure S6d–f). Accordingly, the iDPC technique visualizes the presence of oxygen vacancies in BT‐N but not 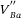
‐$V_{O}^{\cdot \cdot }$ defect dipole as that in B99T, as shown in Figure 3e, f1‐f3 and Figure S8.

The effects of oxygen vacancy migration and defect dipoles on the electric fatigue behavior of electrostrain present a distinct difference. The BT‐N sample exhibits significant continuous degradation under cyclic loading due to oxygen vacancy migration, with a performance decay of over 60% after 10^4^ bipolar cycles at 1 Hz (Figure S9). In contrast, the B99T sample shows only a 20% reduction under the same conditions (Figure S10). Fatigue in BT‐N should be attributed to the high leakage induced by long‐range migration of oxygen vacancies (Figure S11) [25, 26], whereas in B99T the fatigue stems from unstable <110> defect‐dipole alignment in the tetragonal matrix [27]. Under unipolar cyclic loading, B99T promotes the alignment of defect dipoles [2], resulting in a 40% performance enhancement at −20 kV cm^−1^ and 15% at +20 kV cm^−1^. In comparison, BT‐N exhibits a performance decline of over 33% after both unipolar loading cycles. These results highlight the application potential of defect‐dipole‐mediated piezoelectric ceramics.

### 2.2 Self‐Alignment of Defect Dipoles and Electrobending Induced by a Continuous Gradient Distribution of Defect Dipoles

**FIGURE 4:**
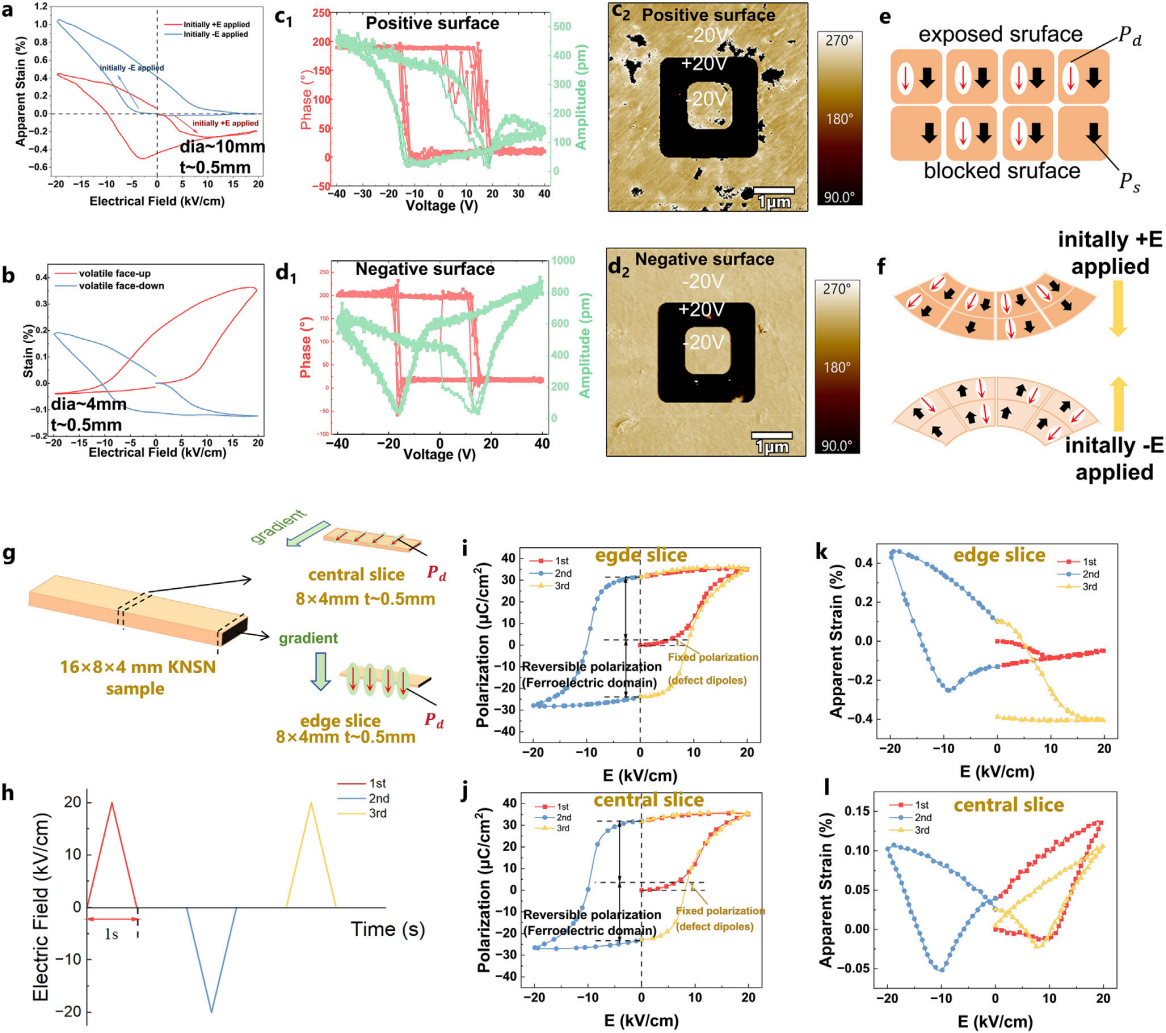
(a) Apparent S‐E curves of 0.5 mm‐thick KNSN ceramics with defect dipole gradient (10 mm in diameter); (b) true S‐E curves of KNSN ceramics; (c_1_‐d_1_) phase angle and piezoelectric amplitude‐voltage curves of the surfaces of poled KNSN sample measured utilizing Piezoelectric force microscopy (PFM) (c_2_‐d_2_) domain writing results for positive surface and negative surface of KNSN sample; (e) illustration of self‐alignment of defect dipoles due to gradient distribution; (f) illustration of poling‐independent bending deformation in KNSN ceramics. (g) Schematic representation of KNSN sample slices; (h) the waveform of the electric field applied during the initial and subsequent electrical measurements of the KNSN samples; (i) and (j) P‐E loops for the edge and central slices during the first electric‐field loading test, respectively; (k) and (l) S‐E curves for the edge and central slices during the first electric‐field loading test, respectively.

As defect dipoles can give rise to enhanced asymmetric strain, the gradient distribution of defect dipoles will induce electrobending deformation in piezoelectric ceramics. In our previous work, we fabricated a B99T/BT bilayer ceramic and confirmed that electrobending occurs in this ceramic due to uneven defect dipole distribution [2]. Different from bending caused by oxygen vacancy migration, the bending deformation in B99T/BT ceramic is poling‐dependent: positively poled sample leads to concave bending at positive applied electric field (+E) and convex bending at −E, while negatively poled sample presents convex bending at +E and concave bending at −E [2]. Owing to the volatility of alkali metals, a continuous gradient distribution of A‐site vacancies and oxygen vacancies is likely to be generated in KNN‐based and NBT‐based ceramics. However, when attempting to verify gradient‐distributed defect‐dipole‐induced electrobending in Sr‐doped KNN‐based ceramics (KNSN), we found that the electrobending in KNSN is poling‐direction‐independent: regardless of whether the initially applied electric field is positive or negative, the apparent electrostrain curves of KNSN consistently show a left‐high/right‐low profile, indicating concave bending under a positive field (+E) and convex bending under a negative field (−E), as depicted in Figure 4a and Figure S12a. Poling‐direction‐independent electrobending in volatile piezoelectric ceramics appears to be in conflict with conventional defect‐dipole alignment models [1, 2], and thus was attributed to oxygen vacancy migration in some studies [6, 8]. To further reconcile the origin of the large apparent strain observed in volatile piezoelectric ceramics, the sample size is reduced to 4 × 4 × 0.5 mm to minimize the contribution from bending deformation. The strain curves of the small KNSN sample remain asymmetric and can be mirror‐inverted upon flipping the sample (Figure 4b; Figure S12b), which is fundamentally different from electro‐deformation caused by oxygen‐vacancy migration (see Notes S1 and S3 for the methods used to differentiate and evaluate the contribution of defect dipoles and oxygen vacancies) [8]. PFM results further confirm that ferroelectric domain switching at both the positive and negative surfaces of the KNSN ceramic is not suppressed (Figure 4c1,c2,d1,d2), indicating that the electrobending observed in KNSN does not originate from oxygen‐vacancy migration. Meanwhile, the amplitude–voltage curve still exhibits an asymmetry similar to that of the macroscopic strain response, as shown in Figure 4c1,d1, suggesting that defect dipoles exist in KNSN and contribute to the electric‐field‐induced strain. The positive and negative surfaces of the sample exhibit “left‐high/right‐low” and “left‐low/right‐high” amplitude responses, respectively, which is supportive of the expected surface response induced by aligned defect dipoles. It should be noted that $V_{K/Na}^{\prime }$‐$V_{O}^{\cdot \cdot }$ defect dipoles neither induce internal bias nor prohibit domain switching [28], so the surface domain switching of the KNSN ceramic cannot exhibit the reinforcement or restriction observed in the B99T ceramic. These results indicate a contribution from defect dipoles in KNSN, yet their orientation appears to be independent of the poling direction. When defect dipoles are distributed in a continuous gradient, they may preferentially align along the gradient direction and remain unchanged under the applied electric field (Figure 4e). As a result, the true strain response shows a left‐low/right‐high profile when the volatilized surface faces upward, and a left‐high/right‐low profile when it faces downward (Figure S12c). If such orientation indeed exists, then for larger samples that are prone to bending, the specimen consistently exhibits concave bending under a positive electric field and convex bending under a negative electric field, regardless of the poling direction, as illustrated in Figure 4f and Figure S12d.

In order to experimentally determine the correlation between defect gradient and alignment of the defect dipole, large KNSN bulks (16 × 8 × 4 mm^3^) were sliced into 8 × 4 × 0.5 mm^3^ pieces, and different slices were selected from the edge and the central region for comparative analysis, as shown in Figure 4g. Volatilization of K/Na elements during sintering creates a continuous gradient in defect concentration from surface to bulk [29]. For the central slice, the defect gradient distribution is predominantly from the top to the bottom edge. Conversely, in the edge slice, the defect dipoles are aligned out‐of‐plane along with the defect gradient. Such a gradient distribution of defect dipoles is validated in Secondary Ion Mass Spectrometry (SIMS) measurements (Figure S13). The P‐E tests were conducted on both central and edge slices; the corresponding electric field waveforms of the applied electric field are shown in Figure 4h, and the results are presented in Figure 4i,j. In our earlier work, a large additional fixed polarization (P*) was attributed to an alignment of defect dipoles [28]. However, a minor P* was detected in both central and edge slices, indicating that the electric field exerts little effect on defect dipole alignment. This response suggests that the defect dipoles were already aligned. The edge slice displays distinctly asymmetric curves and a much larger apparent strain (Figure 4k), indicative of a possible out‐of‐plane alignment of defect dipoles. By comparison, the central slice shows symmetric S–E responses and a small strain, likely because an in‐plane defect‐dipole gradient favors in‐plane alignment, yielding little contribution to the out‐of‐plane electrostrain response (Figure 4l). To further verify the reproducibility of the samples, multiple KNSN specimens were prepared, and S‐E curves of 4 × 4 × 0.5 mm edge slices and central slices were measured, as shown in Figure S14. The results show good consistency. Moreover, when powder‐bed buried sintering was employed to suppress volatilization, defect dipoles in the non‐stoichiometric KNSN samples became aligned with the initially applied electric field, accompanied by a larger remanent polarization (P*), as shown in Figure S15.

**FIGURE 5:**
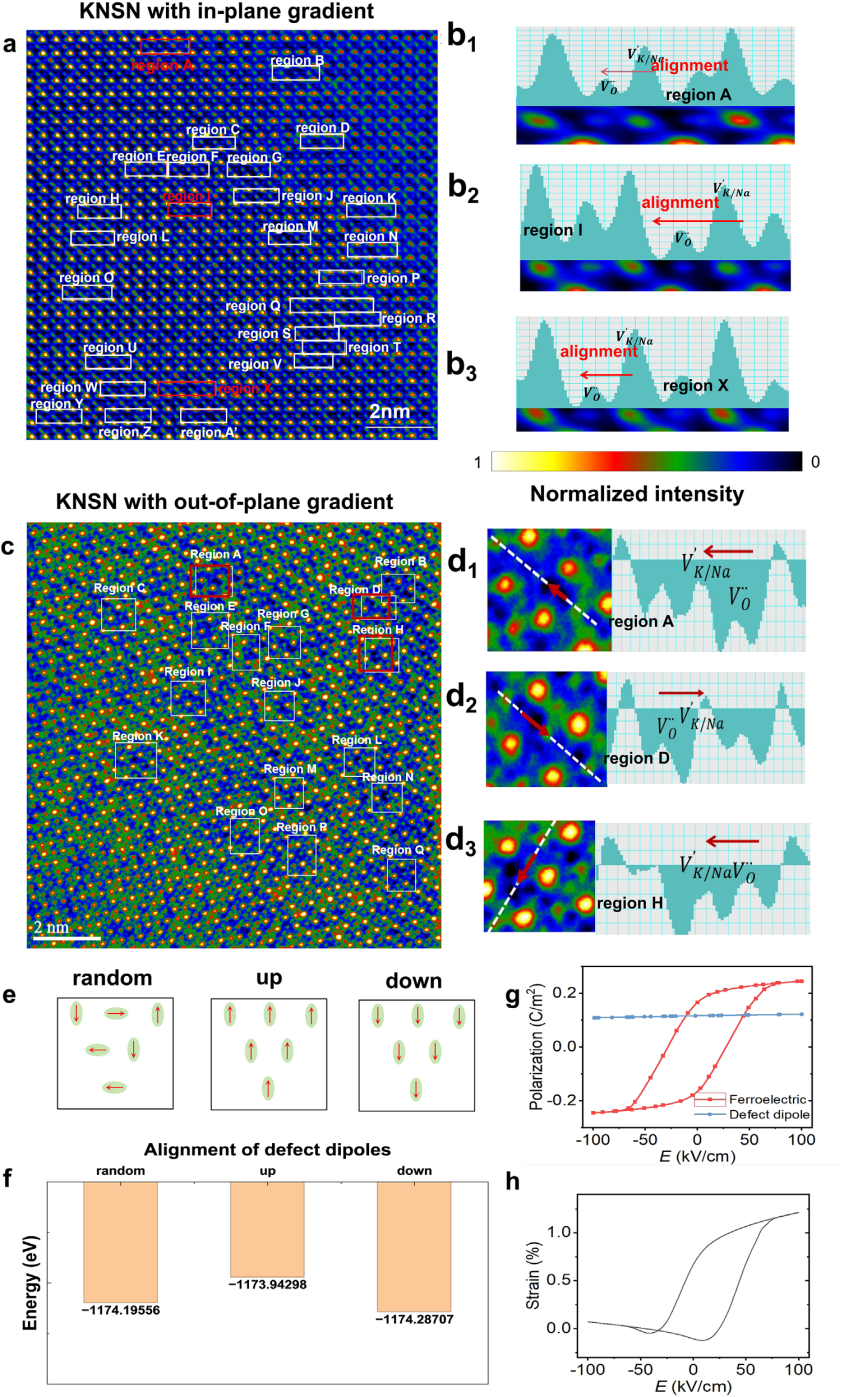
(a) colored iDPC image of KNSN‐in‐plane sample; (b1‐b3) enlarged planar atomic contrast of regions A, I, X in Figure 5a and corresponding line atomic contrast (from left to right); (c) colored iDPC image of KNSN‐out‐of‐plane sample; (d1‐d3) enlarged planar atomic contrast of regions A, D, H in Figure 5c and corresponding line atomic contrast of the white dash line (from left to right); (e) The optimized KNN structures with random‐oriented defect dipoles, upward defect dipoles and downward defect dipoles. Nb: green; O: red; Na: yellow; K: purple. Vacancies: black. (f) Calculated energy for different alignments of defect dipoles. Phase‐field simulation of defect dipole self‐alignment induced by continuous gradient distribution: (g) simulated polarization response of ferroelectric domains and the defect dipole to an electric field; (h) simulated strain response.

We further examined the microscopic defect configurations of the KNSN ceramics. In KNSN, the volatilization of K and Na leads to the formation of K^+^/Na^+^ vacancies, which is confirmed by energy‐dispersive X‐ray spectroscopy elemental analysis in high‐angle annular dark‐field mode (Figure S16). To verify the relationship between the distribution and configuration of defect dipoles, we prepared transmission electron microscopy specimens from a slice with an in‐plane gradient and another slice with an out‐of‐plane gradient, and acquired iDPC‐STEM images to examine the spatial correlation between K/Na vacancies and oxygen vacancies, as shown in Figure S17a–f. For both KNSN‐in‐plane and KNSN‐out‐of‐plane, the intensity distributions at the A‐ and O‐sites exhibit pronounced fluctuations, whereas the B‐site intensity remains relatively uniform, confirming that both samples contain abundant A‐site vacancies and oxygen vacancies (Figure S17g,h). To further compare the spatial correlations among the defects, we selected local regions containing defects in both the KNSN‐in‐plane and KNSN‐out‐of‐plane samples for magnified imaging and detailed line‐scan analyses, as exemplified in the Figure 5a,b1–3,c,d1–3. The paired defect sites that correspond to possible defect dipoles exhibit an almost identical orientation within the projection plane for the KNSN‐in‐plane sample, which accords with our analysis of spontaneous alignment (Figure 5b1–b3) for representatives and Figure S18 for abundant cases; the sample was sectioned from central slices, with an in‐plane vacancy concentration gradient [29]). In contrast, in slices with an out‐of‐plane gradient, the projected orientations of the candidate defect dipoles in iDPC‐STEM images vary from region to region within the projection plane (Figure 5d1–3 for representatives and Figure S19 for abundant cases).

To theoretically rationalize how a continuous defect gradient drives the self‐alignment of defect dipoles, First‐principles calculations were conducted on defect‐gradient‐containing KNN structures with various defect‐dipole orientation directions, as shown in Figure 5e,f (detailed structures are illustrated in Figure S20). The results show that defect dipoles oriented downward (aligned with the gradient) exhibit the lowest total energy, as summarized in Table S1. Phase‐field simulations were also conducted, where a lattice constant gradient and defect dipoles were introduced to mimic the gradient distribution of defect dipoles. The simulations yielded P–E and S–E curves, and spatial polarization maps in various electric fields (Figure 5g; Figure S21). The polarization of ferroelectric domains exhibits a four‐stage evolution, involving switching, saturation, back‐switching, and re‐saturation, which follows the direction of the external field, as depicted in Figure S21. In contrast, the polarization of defect dipoles remains fixed along the direction of the imposed gradient throughout the entire field cycling process. This response demonstrates a self‐alignment of the defect dipole that is driven by the continuous defect concentration gradient. It is worth mentioning that the $V_{K/Na}^{\prime }$‐$V_{O}^{\cdot \cdot }$ defect dipoles do not meet the conditions to establish an internal bias field [28]. Therefore, this self‐alignment does not hinder ferroelectric domain switching, and is hard to reverse by an external electric field. The simulated S–E response shows pronounced asymmetry (Figure 5h), confirming that aligned defect dipoles generate large asymmetric electrostrain.

The self‐alignment of defect dipoles exhibits a high level of stability against electric fields and thermal degradation. The self‐aligned defect dipoles cannot be reversed by high‐temperature poling and aging or even annealing at 500°C, as illustrated in Figure S22a,b. By contrast, in non‐stoichiometric KNSN samples with a much weaker defect‐concentration gradient, defect dipoles can be reversed through high‐temperature poling followed by aging or annealing, as shown in Figure S22c,d.

## 3 Conclusions and Perspective

We have elucidated the mechanisms underpinning defect‐mediated electrostrain and electrobending responses in piezoelectric ceramics. An atomic‐scale visualization of defect dipoles via iDPC‐STEM has confirmed the presence of defect dipoles in Ba_0.99_TiO_2.99_‐based ceramics and self‐alignment of defect dipoles in Sr‐doped KNN ceramics. Isolated oxygen vacancies induce electrobending deformation due to long‐range migration under electric fields, whereas the formation of defect dipoles effectively suppresses this migration, resulting in a true asymmetric electrostrain without macroscopic bending. Moreover, a continuous gradient distribution of defect dipoles triggers self‐alignment, which exhibits ultrahigh stability against electric fields and thermal degradation, accounting for the recently reported poling direction‐independent electrobending responses in ceramics containing volatile elements. Our results reconcile longstanding conflicting interpretations regarding the role of defect dipoles in the electrostrain behavior of piezoelectric materials, providing critical insight into rational defect engineering and paving the way for the development of advanced piezoelectric actuators.

## 4 Materials and Methods

### 4.1 Sample Preparation and Chemical Composition Characterizations

[K_0.485_Na_0.485_Sr_0.03_] NbO_3.01_(KNSN), [K_0.46_Na_0.46_Sr_0.03_] NbO_2.99_(non‐stoichiometric KNSN), BaTiO_3_ (BT), Ba_0.99_TiO_2.99_ (B99T) ceramics were fabricated using a solid‐state reaction method. First, highly pure Na_2_CO_3_(99.9%), K_2_CO_3_(99.5%), SrCO_3_(99.5%), Nb_2_O_5_(99.99%), BaCO_3_(99%), and TiO_2_(99.8%) powders were dried and weighed according to the stoichiometric ratios, followed by ball milling in ethanol for 24 h. After drying, KNSN powders were calcined at 850°C for 6 h in air, and BT/B99T powders were calcined at 900°C for 5 h. After pre‐calcination, the powders were subjected to a secondary ball milling in ethanol for 24 h, followed by drying at 100°C. A 5% poly(vinyl alcohol) solution was then added to the dried powders as a binder. The resultant powders were granulated and pressed into various designed shapes under 200 MPa. Specifically, the KNSN samples were pressed into rectangular plates with dimensions of 20 × 10 × 5 mm, while the other samples were pressed into disc‐shaped pellets with a diameter of 12 mm. The ceramic samples were obtained by sintering in air or N_2_ atmosphere after being calcined at 550°C to remove the binder: KNSN was sintered at 1185°C for 6 h, barely without sacrificial powder to promote the volatilization of K/Na elements, and the non‐stoichiometric KNSN was sintered at 1185°C for 3 h with buried sacrificial powder to suppress the volatilization of K/Na elements. Sacrificial powder is employed to suppress the volatilization of K/Na. In samples prepared without sacrificial powder, the defect concentration at the edges is higher than that at the center, and the composition deviates significantly from the designed stoichiometry. In contrast, samples prepared with sacrificial powder exhibit compositions that are consistent with the designed formulation, as confirmed by inductively coupled plasma (ICP) measurements (Table S2). BT and B99T were sintered at 1350°C for 3 h with no sacrificial powder: the B99T samples were sintered in air, while the BT samples were sintered in N_2_ atmosphere. The BT sample sintered in N_2_ atmosphere is referred to as BT‐N. Rectangular KNSN samples were sliced into 8 × 4 × 0.5 mm pieces, and different slices were selected from the edge and the central region for the subsequent tests. All the pellet samples were mechanically thinned to the desired thickness (1 mm, 0.5 mm or 0.3 mm) and fired with an Ag electrode on both sides for further ferroelectric and electric field‐induced strain tests. For BT and B99T ceramics, high‐temperature poling was conducted. During high temperature poling, samples were heated above the Curie temperature and poled at 20 kV cm^−1^ for 20 min. The electric field is removed only when the sample is cooled below 60°C.

Second Ion Mass Spectrometry (SIMS) measurements (ION TOF ToF SIMS 5–100) were conducted on polished slices cut from the as‐sintered KNSN sample. Inductively coupled plasma‐optical emission spectroscopy (ICP‐OES 730, Agilent) was also employed to ascertain the chemical composition gradient. Prior to this analysis, samples were dissolved in hydrochloric acid using a microwave‐digestion system (MILESTONE START D). X‐ray Photoelectron Spectroscopy (XPS, *ESXCALAB Xi+) was used to evaluate the valence states of the Ti element. The amount of $V_{o}^{\cdot \cdot }$ was qualitatively tested by electron paramagnetic resonance (EPR) at room temperature using an EPR spectrometer (Bruker E580) operating in the X‐band frequency (9.8585 GHz) with a field modulation frequency of 100 kHz. To measure the change in surface oxygen vacancy concentration after poling, the samples were first coated with silver paste at room temperature and then poled at 150°C. After polishing, the silver paste on the surface was removed using acetone, and a 200 µm‐thick section was cut from both the positive and negative surfaces of the sample using a diamond wire saw, followed by EPR measurements.

### 4.2 Electric Properties Measurement

The polarization hysteresis loops and electric‐field‐induced S‐E curves were recorded using a ferroelectric tester (Radiant Precision LC II). Bipolar and unipolar triangular waveforms with frequencies of 1 Hz were employed for P‐E, I‐E, and S‐E curves. The unipolar fatigue at room temperature was evaluated using 100 Hz or 1 Hz and 20 kV cm^−1^ bipolar or unipolar triangular waveforms, and then tested at 1 Hz and 20 kV cm^−1^. The electro‐induced vibration modes of the samples were detected using Scanning Laser Vibration meters (SOPTOP LV‐FSC500) for cross‐validation of the results, in which the samples were connected to copper wires using epoxy conductive silver adhesive and conductive carbon film, to apply an alternating ±20 kV cm^−1^ electric field.

### 4.3 Structure and Microstructure Characterizations

Polished BT‐N, B99T, and KNSN ceramic samples were used for Piezoresponse Force Microscopy (PFM, MFP‐3D, Asylum Research) characterization and domain‐writing experiment. Conductive cantilevers ASYELEC‐01 with tip coatings of Ti/Ir (5/20) were used. The nominal spring constants were 2 N m^−1^ with a fundamental resonance frequency of the free tip‐vibration (non‐contact resonance) of approximately 70 kHz. TEM samples were prepared by a conventional method including mechanical thinning, Ar+‐ion milling, and carbon‐film coating. The atomic‐scale imaging was carried out on a Cs‐corrected Thermo Spectra 200. 200 kV with ultra‐high resolution mode and a convergence/collection semi‐angle of 20 mrad/60‒320 mrad. The iDPC‐STEM imaging was also conducted using the Cs‐corrected Thermo Spectra 200. with a convergence semiangle of 15 mrad, operated at a voltage of 200 kV. The collection angle for the iDPC‐STEM imaging is ∼4–20 mrad. For iDPC‐STEM characterization, all samples were thinned to 10–20 nm to minimize atomic column overlap and enable better visualization of oxygen vacancies. The KNSN samples for iDPC‐STEM were sectioned along the vertical thickness direction to ensure an in‐plane defect gradient.

### 4.4 Phase‐Field Simulation

In the phase‐field model, the microstructure of the ferroelectric material is described by the ferroelectric polarization field **P**(**x**) and the defect dipole polarization field **P** ^def^(**x**) within the simulation system, where **x** is the spatial position vector. The evolution of these variable fields under given external conditions is governed by the time‐dependent Ginzburg‐Landau equations, written as

(1) $$\frac{\partial P}{\partial t}=L^{P}\;\left(-\frac{\delta F}{\delta P}+E^{\mathrm{therm}}\right)$$

(2) $$\frac{\partial P^{\mathrm{def}}}{\partial t}=L^{\mathrm{def}}\;\left(-\frac{\delta F}{\delta P^{\mathrm{def}}}+E^{\mathrm{therm},\mathrm{def}}\right)$$

Equations (1) and (2) describe a relaxation process driven by a minimization of the thermodynamic free energy of the system under given conditions; here *t* is the time and **L** ^P^ and **L** ^def^ are the kinetic coefficients of the ferroelectric polarization and the defect dipole polarization, respectively. *F* is the polarization‐ field‐ dependent part of the free energy formulated as a functional of the **P**(**x**) field, the **P** ^def^(**x**) field, and external conditions including the external electric field, the applied stress, and the temperature, etc., taking a hypothetical uniform zero‐polarization state under the same condition, [**P** (**x**) =  0,  **P** ^def^ (**x**) =  0], as a reference. **E** ^therm^ and **E** ^therm,def^ are random fields arising from thermal fluctuations, which obey a normal distribution with the strength given by the fluctuation‐dissipation theorem [30, 31].

The free energy *F* takes contributions from those described in well‐established phase‐field theories of ferroelectric domains [32] as well as additional contributions from the defect dipole order, written as a sum of the Landau free energy *F*_Landau_ for ferroelectric polarization, the bulk energy *F*_defect_ for defect dipole order, the gradient energy *F*_gradient_, the electrostatic energy *F*_electrostatic_, and the elastic energy *F*_elastic_, i.e.,

(3) $$F=F_{\mathrm{Landau}}+F_{\mathrm{defect}}+F_{\mathrm{gradient}}+F_{\mathrm{electrostatic}}+F_{\mathrm{elastic}}$$

The energy contributions are written as

(4) $$\begin{array}{ccc} F_{\mathrm{Landau}} & = & \int \left(a_{i}{P_{i}}^{2}+a_{ij}{P_{i}}^{2}{P_{j}}^{2}+a_{ijk}{P_{i}}^{2}{P_{j}}^{2}{P_{k}}^{2}\right.\\ & & +\left.a_{ijkl}{P_{i}}^{2}{P_{j}}^{2}{P_{k}}^{2}{P_{l}}^{2}\right)\;dx^{3} \end{array}$$

(5)

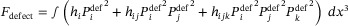

(6) $$F_{\mathrm{gradient}}=\int g_{ijkl}^{P}\frac{\partial P_{i}}{\partial x_{j}}\frac{\partial P_{k}}{\partial x_{l}}\;dx^{3}+\int g_{ijkl}^{\mathrm{def}}\frac{\partial P_{i}^{\mathrm{def}}}{\partial x_{j}}\frac{\partial P_{k}^{\mathrm{def}}}{\partial x_{l}}dx^{3}$$

(7) $$F_{\mathrm{electrostatic}}=\int \left(-\frac{1}{2}E_{i}^{d}P_{i}-\frac{1}{2}E_{i}^{d}P_{i}^{\mathrm{def}}-E_{i}^{\mathrm{ext}}P_{i}-E_{i}^{\mathrm{ext}}P_{i}^{\mathrm{def}}\right)\;dx^{3}$$

(8) $$F_{\mathrm{elastic}}=\int \frac{1}{2}c_{ijkl}\left(\varepsilon_{ij}-\varepsilon_{ij}^{0}\right)\left(\varepsilon_{kl}-\varepsilon_{kl}^{0}\right)\;dx^{3}$$

Indices *i*,  *j*,  *k*,  *l*  =  1, 2, 3 indicate components of a vector or a tensor in a Cartesian coordinate. An Einstein summation convention over repeated indices *i*,  *j*,  *k*, and *l* is employed. *a_i_*, *a_ij_*, *a_ijk_*, and *a_ijkl_* are the Landau coefficients for ferroelectric polarization, *h_i_*, *h_ij_*, and *h_ijk_* are the bulk energy coefficients for defect dipole order, and **g** ^P^ and **g** ^def^ are the gradient energy coefficients for ferroelectric polarization and defect dipole order, respectively. **E** ^d^(**x**) is the depolarization field obtained by solving the electrostatic equilibrium equation at every evolution time step, and **E** ^ext^ is the applied external electric field. **c** is the elastic stiffness tensor, **ε**(**x**) is the strain field obtained by solving the mechanical equilibrium equation, and **ε** ^0^(**x**) is the eigenstrain field given by $\varepsilon_{ij}^{0}=Q_{ijkl}\;\left(P_{k}+P_{k}^{\mathrm{def}}\right)\left(P_{l}+P_{l}^{\mathrm{def}}\right)$, with **Q** being the electrostrictive coefficient. For details of the mechanical and electrostatic equilibrium equations, we refer to [32].

The material constants for the KNN used in the phase‐field simulations are as follows. The Landau coefficients for ferroelectric polarization are taken as *a*_1_ = (*T* − *T*_CW_)  × 3.06 × 10^5^ J m C^−2^K^−1^ where *T*_CW_ is the Curie‐Weiss temperature, *a*_11_ =   − 2.73 × 10^8^ J m^5^C^−4^, *a*_12_ =  10.86 × 10^8^ J m^5^C^−4^, *a*_111_ =  3.04 × 10^9^ J m^9^C^−6^, *a*_112_ =   − 2.73 × 10^9^ J m^9^C^−6^, *a*_123_ =  15.51 × 10^9^ J m^9^C^−6^, *a*_1111_ =  2.40 × 10^10^ J m^13^C^−8^, *a*_1112_ =  0.37 × 10^10^ J m^13^C^−8^, *a*_1122_ =  3.35 × 10^10^ J m^13^C^−8^, and *a*_1123_ =   − 6.20 × 10^10^ J m^13^C^−8^ [33]. The Curie‐Weiss temperature *T*_CW_ takes a spatially inhomogeneous value following a Gaussian distribution with a mean of 357 K and a standard deviation of 200 K is employed to describe a strong perturbation of the energy landscape by the local fluctuation of vacancy species and concentrations within the crystal lattice. The bulk energy coefficients for the defect dipole polarization are taken as *h*_1_ =  6.40 × 10^8^ J m C^−2^, *h*_11_ =   − 2.84 × 10^12^ J m^5^C^−4^, *h*_12_ =   − 3.56 × 10^12^ J m^5^C^−4^, *h*_111_ =  3.16 × 10^15^ J m^9^C^−6^, *h*_112_ =  2.63 × 10^15^ J m^9^C^−6^, and *h*_123_ =  22.72 × 10^15^ J m^9^C^−6^ [3]. The gradient energy coefficients for ferroelectric polarization are $g_{11}^{P}=3.2\times 10^{-11}\;J\;m^{3}C^{-2}$, $g_{12}^{P}=0$, and $g_{44}^{P}=1.6\times 10^{-11}\;J\;m^{3}C^{-2}$ [34]. The gradient energy coefficients for defect dipole polarization are taken as $g_{11}^{\mathrm{def}}=2.0\times 10^{-9}\;J\;m^{3}C^{-2}$, $g_{12}^{\mathrm{def}}=0$, and $g_{44}^{\mathrm{def}}=1.0\times 10^{-9}\;J\;m^{3}C^{-2}$ [18]. The background dielectric constant is taken as $\kappa_{11}^{b}=40$ [35]. The elastic stiffness tensor follows *c*_11_ =  2.30 × 10^11^ J m^−3^, *c*_12_ =  0.90 × 10^11^ J m^−3^, and *c*_44_ =  0.76 × 10^11^ J m^−3^, and the electrostrictive coefficient is taken as *Q*_11_ =  0.16 C^−2^m^4^, *Q*_12_ =   − 0.072 C^−2^m^4^, and *Q*_44_ =  0.084 C^−2^m^4^ [36].

The phase‐field simulations are performed in a quasi‐2D simulation system with a total size of *l*_1_ ×  *l*_2_ =  400 nm × 400 nm, which is discretized into an array of 400 × 400 grids. Periodic boundary conditions of **P**, **P** ^def^, **E**, and **ε** are employed for solving the phase‐field equations. The response of the material microstructure to a slowly varying bipolar electric field loop is simulated, where both the initial ferroelectric polarization **P** and the initial defect dipole polarization **P** ^def^ are taken as random noise with random orientations. The macroscopic polarization loop and strain loop are then obtained by calculating the evolution of the spatial averages of the polarization and the strain within the simulated system.

### 4.5 First‐Principles Calculations

Spin‐polarized first‐principles calculations were performed by the density functional theory (DFT) using the Vienna Ab‐initio Simulation Package (VASP) [37]. The generalized gradient approximation (GGA) with the Perdew‐Burke‐Ernzerhof (PBE) functional was used to describe the electronic exchange and correlation effects [38, 39, 40]. Uniform Γ‐centered k‐points meshes with a resolution of 2π × 0.05 Å^−1^, and Methfessel‐Paxton electronic smearing were adopted for the integration in the Brillouin zone for geometric optimization. The simulation was run with a cutoff energy of 500 eV throughout the computations. The geometry optimization was considered convergent when the electronic energy and Hellmann‐Feynman forces convergence criterion were smaller than 10−5 eV and 0.03 eV Å^−1^, respectively. In the calculation, Grimme's DFT‐D3 [41] methodology was used to describe the dispersion interactions.

## Conflicts of Interest

The authors declare no conflicts of interest.

## Supporting information




**Supporting File 1**: advs75649‐sup‐0001‐SuppMat.docx.

**Supporting File 2**: advs75649‐sup‐0002‐VideoS1.avi.

**Supporting File 3**: advs75649‐sup‐0003‐VideoS2.avi.

## Data Availability

The data that support the findings of this study are available on request from the corresponding author. The data are not publicly available due to privacy or ethical restrictions.
