# Supplementary material for: Asymmetric Electrostrain/Electrobending in Piezoelectric Ceramics: Role of Defect Dipoles or Oxygen Vacancies
Source: Adv Sci (Weinh). 2026 May 10;13(44):e75649. doi: 10.1002/advs.75649 (PMC13335970; doi:10.1002/advs.75649)
Supplement: Supplementary file 1 [file ADVS-13-e75649-s001.docx]

Supplementary Materials for

**Asymmetric Electrostrain/Electrobending in Piezoelectric Ceramics: Role of Defect Dipoles or Oxygen Vacancies**

*Jie Wang, Geng Huangfu, Hongjie Zhang, Tiannan Yang, Zhenhua Ma, Jingsheng Chen, Yun Liu and Yiping Guo**

Corresponding author: ypguo@sjtu.edu.cn (Yiping Guo)

**The PDF file includes:**

Supplementary Notes 1-3

Table S1 and S2

Figs. S1 to S 30

**Other Supplementary Materials for this manuscript include the following:**

Supplementary videos 1 and 2

Supplementary note 1 Distinguishing electrostrain and electrtobending behavior arising from oxygen vacancy migration and defect dipole

Bending effect induced by oxygen vacancy migration is distinctly different from the bending effect caused by the gradient distribution of defect dipoles. For electrobending induced by oxygen vacancy migration, in the absence of defect dipole formation, the intrinsic electrostrain S-E curves are symmetric. Asymmetric apparent S-E curves only emerge when the clamp electrode areas are smaller than the sample and differ between the upper and lower surfaces. This asymmetry arises solely from bending-induced displacement. Because the migration direction of oxygen vacancies depends exclusively on the direction of the applied electric field, the sample orientation (face-up or face-down) and polarization state do not affect the apparent S-E curves. Moreover, when the clamp electrode area exceeds the sample size, the symmetric nature of the intrinsic electrostrain response results in equal contributions from convex and concave bending, producing a symmetrical apparent S-E curve. Oxygen vacancy migration consistently causes convex bending when the electric field is directed from the lower to the upper surface and concave bending when the field direction is reversed. Figure S 23 (A)-(D) illustrates electrostrain measurement of BT-N samples using different clamps. When both the clamp electrode areas exceed the sample size, BT-N sample with diameter: thickness ratio of 10mm:0.2mm exhibits symmetrical apparent S-E curve. When the clamp electrode areas are smaller than the sample and differ between the upper and lower surfaces, BT-N sample with diameter: thickness ratio of 10mm:0.2mm exhibits asymmetrical apparent S-E curve. The BT-N samples were cut into small pieces with dimensions of 4 mm × 4 mm × 0.5 mm to effectively minimize the influence of bending, thereby enabling accurate measurement of the intrinsic strain. Under these conditions, the measured electrostrain curve of the BT-N sample is symmetric and exhibits a relatively small magnitude, indicating that the migration of oxygen vacancies has no significant effect on the macroscopic intrinsic strain of the sample.

For the unique electrostrain behavior induced by defect dipoles, when the defect dipoles are uniformly distributed, the aligned dipoles produce identical asymmetric electrostrain curves under different mechanical constraints, as shown in Figure S 24 (A)-(D). Moreover, the shape of the strain curve changes from “left-high/right-low” to “left-low/right-high” when the alignment direction of the defect dipoles is changed. For electro-induced bending phenomena arising from gradient distributions of defect dipoles, the material exhibits an inherently asymmetric electrostrain response, even when bending deformation is considered. When clamp electrodes larger than the sample surface are applied to specimens with a high diameter-to-thickness ratio (e.g., dia~10 mm and thickness~ 0.5 mm), pronounced apparent strains are observed under both positive and negative electric fields, as shown in Figure S 25 (a) and (b). These strains result from the combined contributions of bending and intrinsic electrostrain, though they differ in magnitude. Flipping the sample from face-up to face-down—while maintaining the same electrode configuration—leads to a change in shape of strain curve from “left-high/right-low” to “left-low/right-high”, confirming the correlation between asymmetric electrostrain and the alignment direction of defect dipoles. When the clamp electrode areas are smaller than the sample and differ between the top and bottom surfaces, the combined effects of bending and asymmetric intrinsic strain lead to an asymmetric apparent electrostrain response, as shown in Figure S 25 (c). In this case, flipping the sample from face-up to face-down does not change the contribution from bending but reverses the contribution from intrinsic electrostrain. As a result, the apparent electrostrain curve shows a significant reduction in magnitude but does not change “left-low/right-high” shape. When the diameter-to-thickness ratio is reduced (e.g., 4 mm × 0.5 mm), bending deformation is significantly suppressed, and asymmetric electrostrain curves still emerge when clamp electrodes exceed the sample area. In this case, flipping the sample from face-up to face-down continues to change the shape of the S–E curve from “left-low/right-high” to “left-high/right-low”, further validating the strong link between asymmetric electrostrain and the directional alignment of defect dipoles, as shown in Figure S25 (d).

In summary, the fundamental difference between bending induced by oxygen vacancy migration and that caused by a defect dipole gradient lies in their influence on the intrinsic macroscopic electrostrain: oxygen vacancy migration does not promote the true electrostrain, whereas defect dipoles can induce an enhanced asymmetric electrostrain response. This distinction can be made by using samples with a small diameter-to-thickness ratio to minimize the influence of bending, and performing face-up/face-down flipping tests to observe whether the asymmetric electrostrain response reverses upon flipping.

**Supplementary note 2 Quantitative analysis of the effects of defect configurations and sample dimensions on the macroscopically measured strain.**

To quantitatively evaluate the influence of different defect configurations on the apparent strain, finite-element simulations were performed using COMSOL. The elastic compliance matrix and electromechanical coupling matrix were adopted from the material library for Barium Titanate. A piezoelectric coefficient of d_33_*=500pm/V was assigned to represent normal BT ceramics, while reduced coefficients (d_33_* and d_31_*=0pm/V) were used to simulate the oxygen-vacancy-enriched BT surface layer. Based on these parameters, geometries with a diameter of 10 mm and varying thicknesses were constructed to quantitatively analyze the effect of surface oxygen-vacancy migration on the apparent strain.

In the absence of an oxygen-vacancy-enriched layer, the simulated BT ceramics exhibit a strain of ~0.1% under an electric field of 20 kV/cm for thicknesses of 1 mm, 0.5 mm, and 0.3 mm, consistent with the behavior of unpoled B99T ceramics, as shown in Figure S2 and Figure S26. After poling, the strain in the direction parallel to the poling field increases by ~0.05%, indicating the contribution of approximately 1% aligned defect dipoles to the electrostrain.

Previous studies have shown that oxygen-vacancy migration can lead to the formation of a micron-scale enriched layer near the ceramic surface (Scientific Reports 2015, 5, 14576.) (Applied Physics Letters 2025, 126 (20), 202901.). Accordingly, a 0.01 mm-thick oxygen-vacancy-enriched layer was introduced into the models with thicknesses of 1 mm, 0.5 mm, and 0.3 mm to quantitatively simulate its effect on electrobending-induced apparent strain. Upon introducing this layer, the ceramics exhibit concave bending under a positive electric field and convex bending under a negative electric field, with the bending effect becoming more pronounced as the thickness decreases, as shown in Figure S27.

For the 1 mm-thick sample, the bending effect is weak, yielding apparent strains of 0.10% and 0.11% under +20 kV/cm and −20 kV/cm, respectively. For the 0.5 mm-thick sample, the bending leads to an apparent strain of 0.10% at +20 kV/cm and 0.20% at −20 kV/cm. In the 0.3 mm-thick sample, the bending effect becomes significant, with the apparent strain remaining ~0.10% at +20 kV/cm but increasing to 0.60% at −20 kV/cm. These results quantitatively demonstrate the impact of a 10 μm-thick oxygen-vacancy-enriched layer on the apparent strain, in good agreement with the experimental observations (Figure S2).

Based on the apparent strain measured at different thicknesses, the corresponding curvature can be calculated as	$R^{2}={(R-d+\delta)}^{2}+r^{2}$	(Eq. S1)

, where R is curvature radius, d is measured displacement, δis piezoelectric displacement and r is approximately radius of the sample. The thickness dependence of curvature for both BT–N and KNSN ceramics under an applied electric field of 20 kV/cm is presented in the figure (thickness-dependent measured electrostrain is obtained from our previous work (*Advanced Materials* 2024, 36, 2404682.)), which quantitatively captures the electrobending behavior arising from oxygen-vacancy migration and from the gradient distribution of defect dipoles at different sample thicknesses, as shown in Figure S28.

**Supplementary note 3 Evaluating contribution of defect dipoles to electrostrain and electrobending in bending samples**

The bending deformation of the material can be quantified using a scanning laser vibrometer and sample-flipping electrostrain testing. For convex deformed sample, the displacement at center point is d_1_, and the displacement at edge point is d_2_; for concave deformed sample, the displacement at edge point is d_3_, the displacement at center point is d_4_, as shown in Figure S29 (a)-(b). These displacements should satisfy:

$d_{1}=\delta_{s}^{-}+d_{bending}^{-}$	(Eq. S2)

$d_{2}=\delta_{s}^{-}-d_{bending}^{-}$	(Eq. S3)

$d_{3}=\delta_{s}^{+}+d_{bending}^{+}$	(Eq. S4)

$d_{4}=\delta_{s}^{+}-d_{bending}^{+}$	(Eq. S5)

where $\delta_{s}^{-}$ and $\delta_{s}^{+}$ are displacement caused by electrostrain under negative and positive electric field; $d_{bending}^{-}$ and $d_{bending}^{+}$ are displacement caused by bending deformation under negative and positive electric field. According to the sample displacements obtained from scanning laser vibrometer (Figure S29 (c)-(f)), the displacements satisfy:

d_1_-d_2_=d_3_-d_4_.	(Eq. S6)

Therefore, displacements caused by bending deformation under negative and positive electric field are equal according to equation (S2) -(S6):

$d_{bending}^{-}=d_{bending}^{+}$	(Eq. S7)

Convert equation (S7) into (S2) -(S5), we can obtain that:

$d_{3}-d_{1}=d_{4}-d_{2}=\delta_{s}^{+}-\delta_{s}^{-}$,	(Eq. S8)

where $\delta_{s}^{+}-\delta_{s}^{-}$ can reflect the electrostrain value induced by lattice stretching and contracting. Based on the results from scanning laser vibrometer, the electrostrain value difference of KNSN2-Sn is about 0.28% at bipolar 15kV/cm, with effective bipolar d_33_* of 1833pm/V.

For measurement of electrostrain using ferroelectric analyzer, as the surface area of fixture is larger than sample for both upper and lower contact surface, the detector can only measure d_1_ and d_3_, as shown in Figure S30 (a) and (c). The S-E curves of 10:0.5mm and 4:0.5mm KNSN2-Sn samples under bipolar 15kV/cm are measured to compare with the results from scanning laser vibrometer (15kV/cm is used because 750V is the maximum applied electric field for scanning laser vibrometer). The sample in ferroelectric analyzer measurement is in completely clamping state with a large preload, while sample in scanning laser vibrometer measurement is in free deformation state. Consequently, the bending deformation is smaller in ferroelectric analyzer testing due to the preload. In 10:0.5mm sample, an evident d_bending_ still exists in the measured S-E curve with a preload, leading to much larger apparent strain (0.6%) compared with true electrostrain (0.28%). Through calculating d_3_-d_1_, $\delta_{s}^{+}-\delta_{s}^{-}$ is 0.27%, consistent with results from scanning laser vibrometer. In 4:0.5mm sample, the weak bending deformation is further restrained with a preload, so d_bending_ cannot be observed in the measured S-E curve, which means S-E curve reflects the true electrostrain of the sample. The $\delta_{s}^{+}-\delta_{s}^{-}$ value obtained from S-E curve of 4:0.5mm sample is 0.28%, also consistent with the results from scanning laser vibrometer. Therefore, the $\delta_{s}^{+}-\delta_{s}^{-}$ value refers to true electrostrain value of the sample.

**TBALE S1** The calculated energy of random, up and down types (unit: eV).

|  | random | up | down |
| --- | --- | --- | --- |
| energy (eV) | -1174.19555999 | -1173.94297670 | -1174.28706571 |

**TABLE S2** Composition determination of KNSN ceramic powders

| Position | Element | Mass fraction (mg/kg) | Molar mass (g/mol) | Atomic content (mol/kg) | Stoichiometry* | Designed stoichiometry | $\boldsymbol{V}_{\boldsymbol{K}/\boldsymbol{Na}}^{'}$ content | $\mathbf{V}_{\mathbf{O}}^{\cdot\cdot}$ content† |
| --- | --- | --- | --- | --- | --- | --- | --- | --- |
| Edge slice of 6h-sintered KNSN | K | 97623.278627451 | 39.1 | 2.4968 | 0.445985218 | 0.4850 | 0.074 | 0.022 |
|  | Na | 57913.20426 | 22.99 | 2.5191 | 0.449968856 | 0.4850 |  |  |
|  | Nb | 520133.4691 | 92.91 | 5.5983 | 1 | 1.0000 |  |  |
|  | Sr | 16140.12618 | 87.62 | 0.1842 | 0.03290391 | 0.0300 |  |  |
| Central slice of 6h-sintered KNSN | K | 98466.88373 | 39.1 | 2.5183 | 0.460390242 | 0.4850 | 0.0467 | 0.0084 |
|  | Na | 58207.82098 | 22.99 | 2.5319 | 0.462865748 | 0.4850 |  |  |
|  | Nb | 508219.9926 | 92.91 | 5.4700 | 1 | 1.0000 |  |  |
|  | Sr | 16148.12554 | 87.62 | 0.1843 | 0.033692368 | 0.0300 |  |  |
| Upper surface of 3h-sintered KNSN | K | 98243.27691 | 39.1 | 2.5126 | 0.457554681 | 0.4600 | 0.0533 | 0.0116 |
|  | Na | 57971.22469 | 22.99 | 2.5216 | 0.459187889 | 0.4600 |  |  |
|  | Nb | 510203.8789 | 92.91 | 5.4914 | 1 | 1.0000 |  |  |
|  | Sr | 15574.25572 | 87.62 | 0.1777 | 0.032368381 | 0.0300 |  |  |
| Lower surface of 3h-sintered KNSN | K | 96940.03795 | 39.1 | 2.4793 | 0.458982331 | 0.4600 | 0.0509 | 0.0104 |
|  | Na | 57138.62898 | 22.99 | 2.4854 | 0.460108635 | 0.4600 |  |  |
|  | Nb | 501870.6026 | 92.91 | 5.4017 | 1 | 1.0000 |  |  |
|  | Sr | 15280.64557 | 87.62 | 0.1744 | 0.032285535 | 0.0300 |  |  |

*Atomic contents of Nb are taken as the standard when calculating the stoichiometric numbers.

†$\mathbf{V}_{\mathbf{O}}^{\boldsymbol{\cdot\cdot}}$ contents are calculated based on the principle of electricity valence balance, and excess O atoms caused by Sr doping are taken into account.

**Figure S 1 (**a) XRD patterns of the piezoelectric ceramics in this work; (b)illustration of electrostrain measurement setup. All ceramics are phase-pure without detectable secondary phases, exhibiting diffraction patterns characteristic of a single perovskite structure. Among them, the KNSN-based ceramics show a (200)/(020) peak-splitting intensity ratio close to 2:1, indicative of an orthorhombic phase, whereas the BT-based ceramics display a (200)/(020) splitting ratio between 1:1 and 1:2, suggesting a stronger tendency toward a tetragonal phase at room temperature.

**Figure S 2** Thickness-dependent electric properties measurement of B99T and BT-N samples: (a) P-E loops of unpoled BT-N samples with different thicknesses; (b) S-E curves of unpoled BT-N samples with different thicknesses;(c) P-E loops of poled BT-N samples with different thicknesses; (d) S-E curves of poled BT-N samples with different thicknesses; (e) P-E loops of unpoled B99T samples with different thicknesses; (f) S-E curves of unpoled B99T samples with different thicknesses;(g) P-E loops of poled B99T samples with different thicknesses; (h) S-E curves of poled B99T samples with different thicknesses. In the BT–N sample, oxygen vacancies migrate toward the surface, leading to suppressed ferroelectric domain switching at the negative surface. As the sample becomes thinner, the increased surface-to-volume ratio results in a reduction in polarization intensity, while the bending effect gives rise to an increased apparent strain. In contrast, in the B99T sample, defect dipoles effectively inhibit oxygen-vacancy migration. After poling, the aligned defect dipoles generate an internal bias field and induce asymmetric strain, the magnitude of which does not vary significantly with thickness.

**Figure S 3** (a)-(b) Illustration of defect distribution in virgin and poled BT-N samples;(c1)-(c3) phase angle and piezoelectric amplitude-voltage curves of the virgin BT-N sample, negative surface and positive surface of BT-N sample, respectively; (d1) -(d3) PFM results for the virgin BT-N, surface near negative electrode of poled B99T, and surface near positive electrode of poled BT-N, respectively; (e1) -(e3) domain writing results for the virgin BT-N, surface near negative electrode of poled BT-N, and surface near positive electrode of poled BT-N, respectively.

**Figure S 4** (a)-(b) Illustration of defect distribution in virgin and poled B99T samples;(c1)-(c3) phase angle and piezoelectric amplitude-voltage curves of the virgin B99T sample, negative surface and positive surface of B99T sample, respectively; (d1) -(d3) PFM results for the virgin B99T, surface near negative electrode of poled B99T, and surface near positive electrode of poled B99T, respectively; (e1) -(e3) domain writing results for the virgin B99T, surface near negative electrode of poled B99T, and surface near positive electrode of poled B99T, respectively.

**Figure S 5 The imaging principle of iDPC (integrated Differential Phase Contrast) technique.** The centroid of the convergent beam diffraction (CBED) pattern shifts across different regions of the sample, and both the direction and magnitude of this shift are linearly correlated with the distribution of the sample’s projected internal potential. By scanning the sample in STEM mode using a segmented detector, the centroid shifts in both the X and Y directions can be recorded. Performing a two-dimensional integration of this data yields an image that approximately represents the projected internal potential distribution of the sample. Since the internal potential distribution is directly related to the type and precise positions of atoms within the sample, this method enables the visualization of individual atomic positions.

**Figure S 6** (a)Normalized intensity distribution of A-site atoms in iDPC-STEM image of the B99T sample; (b)normalized intensity distribution of B-site atoms in iDPC-STEM image of the B99T sample; (c)normalized intensity distribution of O-site atoms in iDPC-STEM image of the B99T sample; (d)Normalized intensity distribution of A-site atoms in iDPC-STEM image of the BT-N sample; (e)normalized intensity distribution of B-site atoms in iDPC-STEM image of the BT-N sample; (f)normalized intensity distribution of O-site atoms in iDPC-STEM image of the BT-N sample.

**Figure S 7** IDPC-STEM image of the B99T sample for (A) gray-scale contrast and (B) temperature-colored contrast; (C) planar atomic contrast of regions A-R and corresponding atomic contrast of the diagonal lines in these regions. iDPC scanning transmission electron microscopy (STEM) linearly images the projected electrostatic potentials of atomic columns, generating a contrast mechanism that is almost proportional to atomic number (Z) and is sensitive to lighter elements such as oxygen. Certain Ba and O sites exhibit distinctly lower contrast compared with neighboring atomic columns (identified by dashed boxes), and consistently appear in coupled pairs. In the image, twenty distinct regions containing oxygen vacancies can be clearly identified, labeled as regions A to T. Among them, nineteen regions exhibit oxygen vacancies adjacent to Ba²⁺ vacancies, indicating the formation of $\boldsymbol{V}_{\boldsymbol{Ba}}^{\boldsymbol{''}}$**-**$\boldsymbol{V}_{\boldsymbol{O}}^{\boldsymbol{\cdot\cdot}}$ defect dipoles. In region S, the oxygen vacancy is not adjacent to any Ba^2+^ vacancy. However, a Ba^2+^ vacancy is still present nearby. Under an external electric field, the migration of the oxygen vacancy in region S may lead to the formation of new defect dipoles associated with the Ba^2+^ nearby, which could hinder the long-range migration of oxygen vacancies.

**Figure S 8** iDPC-STEM image of the BT-N sample for (A) gray-scale contrast and (B) temperature-colored contrast; (C) planar atomic contrast of regions A-R and corresponding atomic contrast of the diagonal lines in these regions. All the oxygen vacancies observed in this sample appear as isolated oxygen vacancies.

**Figure S 9** Fatigue resistance tests of BT-N samples. (A) Ferroelectric hysteresis loops after bipolar fatigue at ±20 kV/cm at 100Hz; (B) Apparent S–E curves after bipolar fatigue at ±20 kV/cm at 100Hz; (C) Ferroelectric hysteresis loops after bipolar fatigue at ±20 kV/cm at 1Hz; (D) Apparent S–E curves after bipolar fatigue at ±20 kV/cm at 1Hz; (E) Apparent unipolar S–E curves after unipolar fatigue at –20 kV/cm at 1Hz; (F) Apparent unipolar S–E curves after unipolar fatigue at +20 kV/cm at 1Hz.

**Figure S 10** Fatigue resistance tests of B99T samples. (A) Ferroelectric hysteresis loops after bipolar fatigue at ±20 kV/cm at 100Hz; (B) Apparent S–E curves after bipolar fatigue at ±20 kV/cm at 100Hz; (C) Ferroelectric hysteresis loops after bipolar fatigue at ±20 kV/cm at 1Hz; (D) Apparent S–E curves after bipolar fatigue at ±20 kV/cm at 1Hz; (E) Apparent unipolar S–E curves after unipolar fatigue at –20 kV/cm at 1Hz; (F) Apparent unipolar S–E curves after unipolar fatigue at +20 kV/cm at 1Hz.

**Figure S11** Current density curves during fatigue testing for (a) B99T ceramic and (b) BT-N ceramic.

**Figure S 12** (A) Illustration of electrostrain measurement setup for Φ10mm×0.5mm KNSN samples; (B) illustration of electrostrain measurement setup for 4mm×4mm×0.5mm KNSN samples; (c)illustration of asymmetric electrostrain due to gradient-aligned defect dipoles; (d) illustration of bending deformation due to gradient-aligned defect dipoles.

**Figure S13** Second Ion Mass Spectrometry (SIMS) measurements for different slices from KNSN sample. To demonstrate that the 16 × 8 × 4 mm KNSN sample possesses a gradient distribution of K/Na vacancies from the top to the bottom and from the edges toward the center, we extracted three slices: slice 1# from the edge region, slice 2# from the middle region, and slice 3# from the bottom. Slice 1#, taken from the sample edge, is entirely exposed to the exposed surface; hence, it does not show an in-plane gradient but exhibits overall lower K/Na content than the central slice, indicating more severe volatilization and a higher concentration of K/Na vacancies in this region. Slice 2#, located in the middle of the sample, shows higher overall K/Na content compared with the edge slice. Because the upper side of slice 2# is adjacent to the exposed surface while the lower side is adjacent to the blocking surface, this slice exhibits an in-plane gradient with decreasing K/Na vacancy concentration from top to bottom. Slice 3#, taken from the bottom region, is entirely adjacent to the blocking surface and therefore shows higher K/Na content than the edge slice; moreover, the region near the sample edge also displays a vacancy gradient, indicating that the exposed surface at the edges induces an out-of-plane gradient distribution in the edge region.

**Figure S14** Reproducibility of the samples: (a) S-E curves for 4×4×0.5mm edge slices from different KNSN sample when volatile surface facing up; (b) S-E curves for 4×4×0.5mm edge slices from different KNSN sample when volatile surface facing sown; (c) S-E curves for 4×4×0.5mm central slices from different KNSN sample; (d) The maximum strain of different samples at −20 kV/cm and +20 kV/cm, with error bars.

**Figure S15** (a)The waveform of the electric field applied during the initial and subsequent electrical measurements of the KNSN samples when the first applied electric field is in positive direction; (b) P–E curve with the first applied electric field in the positive direction; (c) S–E curve with the first applied electric field in the positive direction; (d)The waveform of the electric field applied during the initial and subsequent electrical measurements of the KNSN samples when the first applied electric field is in negative direction; (e) P–E curve with the first applied electric field in the negative direction; (f) S–E curve with the first applied electric field in the negative direction.

**Figure S 16** The atomic-scale HAADF image marked with normalized A site intensity by color (A) and associated EDS analysis for K element (B) and Na element (C). A large number of K^+^/Na^+^ vacancies can be observed by the intensity distribution maps.

**Figure S17** (a)Normalized intensity distribution of A-site atoms in iDPC-STEM image of the KNSN-in-plane sample; (b)normalized intensity distribution of B-site atoms in iDPC-STEM image of KNSN-in-plane sample; (c)normalized intensity distribution of O-site atoms in iDPC-STEM image of the KNSN-in-plane sample; (d)Normalized intensity distribution of A-site atoms in iDPC-STEM image of the KNSN-out-of-plane sample; (e)normalized intensity distribution of B-site atoms in iDPC-STEM image of KNSN-out-of-plane sample; (f)normalized intensity distribution of O-site atoms in iDPC-STEM image of the KNSN-out-of-plane sample; (g) Scatter plots and boxplots of the intensity distributions at the A-, B-, and O-sites in the KNSN-in-plane sample; (h) Scatter plots and boxplots of the intensity distributions at the A-, B-, and O-sites in the KNSN-out-of-plane sample;

**Figure S 18** IDPC-STEM image of the KNSN-in-plane sample for (A) gray-scale contrast and (B) temperature-colored contrast; (C) enlarged planar atomic contrast of regions A-A’ and corresponding line atomic contrast from left to right. (D)-(F) atomic contrast and intensity profile for another region of KNSN-in-plane. Due to the difference in valence states, the concentrations of K/Na vacancies and oxygen vacancies can vary significantly, resulting in complex defect configurations observed utilizing iDPC technique. In addition to the dominant binary $\boldsymbol{V}_{\boldsymbol{K/Na}}^{\boldsymbol{'}}$**-**$\boldsymbol{V}_{\boldsymbol{O}}^{\boldsymbol{\cdot\cdot}}$ defect dipoles, ternary configurations such as $\boldsymbol{V}_{\boldsymbol{K/Na}}^{\boldsymbol{'}}$**-**$\boldsymbol{V}_{\boldsymbol{O}}^{\boldsymbol{\cdot\cdot}}$**-**$\boldsymbol{V}_{\boldsymbol{K/Na}}^{\boldsymbol{'}}$ can also be observed. Among the 27 regions containing oxygen vacancies, 19 $\boldsymbol{V}_{\boldsymbol{K/Na}}^{\boldsymbol{'}}$**-**$\boldsymbol{V}_{\boldsymbol{O}}^{\boldsymbol{\cdot\cdot}}$ defect dipoles and 7 ternary defect configurations can be observed, indicating that defect dipoles constitute the dominant defect configuration. Among these defect dipoles, except for the ternary types without clear alignment, all the binary $\boldsymbol{V}_{\boldsymbol{K/Na}}^{\boldsymbol{'}}$**-**$\boldsymbol{V}_{\boldsymbol{O}}^{\boldsymbol{\cdot\cdot}}$ defect dipoles exhibit a well-defined alignment. Since the KNSN sample was sectioned along its thickness direction, an in-plane defect gradient is expected. The observed alignment of defect dipoles is consistent with the direction of this gradient. Only 3 of the 27 regions contain isolated oxygen vacancies. These isolated vacancies may be captured by other K^+^/Na^+^ vacancies during migration or hopping processes to form new defect dipoles as abundant K^+^/Na^+^ vacancies exist, which verified by EDS analysis in Figure S7. For regions selected from (D)-(F), there are 22 regions with alignment defect dipoles in total 30 representative regions that containing oxygen vacancies, also evidencing the aliment of defect dipoles in gradient direction.

**Figure S 19** iDPC-STEM image of the KNSN-out-of-plane sample for (A) gray-scale contrast and (B) temperature-colored contrast; (C) enlarged planar atomic contrast of regions A-Q and corresponding line atomic contrast of the white line from left to right. In KNSN-out-of-plane sample, Defect dipoles with different projected orientations were observed, confirming that the sample shows no preferential alignment in this projection plane.

**Figure S20** The optimized KNN structures with (a) random-oriented defect dipoles, (b) upward defect dipoles and (c) downward defect dipoles. Nb: green; O: red; Na: yellow; K: purple. Vacancies:black.

**Figure S 21** **Phase-field simulation of defect dipole self-alignment induced by gradient distribution.** (a) -(d) Polarization distribution of ferroelectric domains at points A-D shown in the P-E curves in Figure 3 (c), respectively.

**Figure S 22** Electrostrain tests for poled samples and re-poled samples after ageing or annealing:(A) volatile KNSN before and after ageing experiment; (B) volatile KNSN sample before and after annealing experiment; (C) Non-stoichiometric KNSN before and after ageing experiment; (D) Non-stoichiometric KNSN sample before and after annealing experiment. The volatilization of K/Na is effectively suppressed in non-stoichiometric KNSN ceramics through powder-buried sintering, resulting in a relatively uniform distribution of defect dipoles. Under such conditions, the alignment of defect dipoles can be reversibly switched by applying an external poling electric field. In contrast, when the KNSN ceramics are exposed during sintering, enhanced volatilization leads to a gradient distribution of defect dipoles. This non-uniform distribution induces a self-aligned configuration of defect dipoles, which becomes resistant to reorientation under external electric fields.

**Figure S 23** Electrostrain measurement of BT-N samples with different clamps. (A) illustration of different clamps; apparent S-E curves measured at (B) test mode I; (C) test mode ii and (D) test mode iii, respectively.

**Figure S 24** Electrostrain measurement of B99T samples with different clamps. (A) illustration of different clamps; apparent S-E curves measured at (B) test mode I; (C) test mode ii and (D) test mode iii, respectively.

**Figure S 25** Electrostrain measurement of Sr-Sn co-doped KNN ceramic (KNSNS) samples with different clamps. (a) illustration of different clamps; apparent S-E curves measured at (b) test mode I; (c) test mode ii and (d) test mode iii, respectively.

**Figure S26**. Finite-element simulation of the thickness dependence of electrostrain in BT ceramics in the absence of bending: (a) 1 mm thickness; (b) 0.5 mm thickness; (c) 0.3 mm thickness.

**Figure S27**. Finite-element simulation of the thickness dependence of electrostrain in BT ceramics with a 0.01mm-thick layer with accumulated oxygen vacancies: (a) and (b) 1 mm thickness; (c) -(d) 0.5 mm thickness; (e)-(f) 0.3 mm thickness.

**Figure S28** Curvature estimation of KNSN and BT-N samples with different thickness

**Figure S 29** Quantify the bending effect of KNSNS ceramics. (a) and (b) schematic illustration of bending measurement using electrodes with larger areas than ceramic sample; (c) and (d) bending deformation and displacement-electric field curves measured by scanning laser vibrometry for a sample with a diameter-to-thickness ratio of 10:0.5 mm, illustrating displacement variations at the center and edge points; (e) and (f) Bending deformation and displacement-electric field curves obtained by scanning laser vibrometry for a sample with a diameter-to-thickness ratio of 4:0.5 mm, depicting displacement variations at the center and edge points.

**Figure S 30** (a) Illustration of deformation measurement for KNSNS sample with diameter: thickness=10:0.5mm; (b) apparent S-E curves for KNSNS sample with diameter: thickness=10:0.5mm； (c)illustration of deformation measurement for KNSNS sample with diameter: thickness=4:0.5mm; (d) S-E curves for KNSNS sample with diameter: thickness=4:0.5mm.
